# Thoracic aortic aneurysm and atrial fibrillation: clinical associations with the risk of stroke from a global federated health network analysis

**DOI:** 10.1007/s11739-022-03184-6

**Published:** 2023-01-14

**Authors:** Riccardo Proietti, José Miguel Rivera-Caravaca, Stephanie Lucy Harrison, Benjamin James Roy Buckley, Raquel López-Gálvez, Francisco Marín, Timothy Fairbairn, Jillian Madine, Riaz Akhtar, Paula Underhill, Mark Field, Gregory Yoke Hong Lip

**Affiliations:** 1 Liverpool Centre for Cardiovascular Science at University of Liverpool, Liverpool John Moores University and Liverpool Heart & Chest Hospital, Liverpool, United Kingdom; 2 Department of Cardiology, Hospital Clínico Universitario Virgen de La Arrixaca, University of Murcia, Instituto Murciano de Investigación Biosanitaria (IMIB-Arrixaca), CIBERCV, Murcia, Spain; 3 Faculty of Nursing, University of Murcia, Murcia, Spain; 4 Department of Cardiovascular and Metabolic Medicine, Institute of Life Course and Medical Sciences, University of Liverpool, Liverpool, UK; 5 Department of Biochemistry and Systems Biology, Institute of Systems, Molecular and Integrative Biology, University of Liverpool, Liverpool, UK; 6 Department of Mechanical, Materials and Aerospace Engineering, School of Engineering, University of Liverpool, Liverpool, L69 3GH UK; 7 TriNetX LLC, London, UK

**Keywords:** Atrial fibrillation, Thoracic aortic aneurysm, Stroke, Risk factors

## Abstract

**Background:**

An association with aortic aneurysm has been reported among patients with atrial fibrillation (AF). The aims of this study were to investigate the prevalence of thoracic aorta aneurysm (TAA) among patients with AF and to assess whether the co-presence of TAA is associated with a higher risk of adverse clinical outcomes.

**Methods and results:**

Using TriNetX, a global federated health research network of anonymised electronic medical records, all adult patients with AF, were categorised into two groups based on the presence of AF and TAA or AF alone. Between 1 January 2017 and 1 January 2019, 874,212 people aged ≥ 18 years with AF were identified. Of these 17,806 (2.04%) had a TAA. After propensity score matching (PSM), 17,805 patients were included in each of the two cohorts. During the 3 years of follow-up, 3079 (17.3%) AF patients with TAA and 2772 (15.6%) patients with AF alone, developed an ischemic stroke or transient ischemic attack (TIA). The risk of ischemic stroke/TIA was significantly higher in patients with AF and TAA (HR 1.09, 95% CI 1.04–1.15; log-rank *p* value < 0.001)

The risk of major bleeding was higher in patients with AF and TAA (OR 1.07, 95% CI 1.01–1.14), but not significant in time-dependent analysis (HR 1.04, 95% CI 0.98–1.10; log-rank *p* value = 0.187),

**Conclusion:**

This retrospective analysis reports a clinical concomitance of the two medical conditions, and shows in a PSM analysis an increased risk of ischemic events in patients affected by TAA and AF compared to AF alone.

**Supplementary Information:**

The online version contains supplementary material available at 10.1007/s11739-022-03184-6.

## Introduction

A thoracic aortic aneurysm (TAA) is a localized dilatation of the ascending and thoracic aorta that can lead to dissection and rupture of the vessel wall [1, 2]. The aortic aneurysm may silently progress with one in two cases being completely asymptomatic [1, 2], and indeed diagnosed incidentally during imaging studies performed for other clinical conditions. The first clinical manifestation may occur as an acute event, either aortic dissection, which associates with a high-risk for mortality or cardiovascular event [1].

Recent observational studies have reported a high prevalence of aortic aneurysms among patients with AF, which is the most common cardiac arrhythmia worldwide [3, 4]. However, the clinical significance of concomitant AF and aortic aneurysms remains undetermined. More specifically, the added risk for cerebrovascular events by the concomitant presence of aneurysms of the aorta in patients with AF is unknown. Currently, indication to oral anticoagulation (OAC) therapy in patients with AF in most guidelines is based on risk stratification built on the pattern of comorbidities and summarized in clinical risk scores, such as the CHA_2_DS_2_-VASc score [5, 6]. In this score, the ‘V’ component has been framed to include myocardial infarction (including significant coronary artery disease on cardiac imaging), peripheral vascular diseases and the presence of atherosclerotic aortic plaque [7]. Nevertheless, diseases of the aorta such as aortic aneurysms are not formally considered in the ‘V’ criterion of the CHA_2_DS_2_-VASc score [6].

In this study using a global federated database of electronic health records, amongst patients with AF, the aims were to examine 1) the prevalence of TAA; and 2) associations between TAA and risk of ischemic stroke, systemic thromboembolic events and major bleeding.

## Methods

We used TriNetX, a global federated health research network with real-time updates of anonymised electronic medical records (EMRs). The network includes healthcare organisations (HCOs, academic medical centres, specialty physician practices and community hospitals), with data for > 80 million patients predominately based in the United States. To comply with legal frameworks and ethical guidelines guarding against data re-identification, the identity of participating HCOs and their individual contribution to each dataset are not disclosed. As a federated research network, studies using the TriNetX health research network do not require ethical approval as no patient identifiable information is received.

**Fig. 1:**
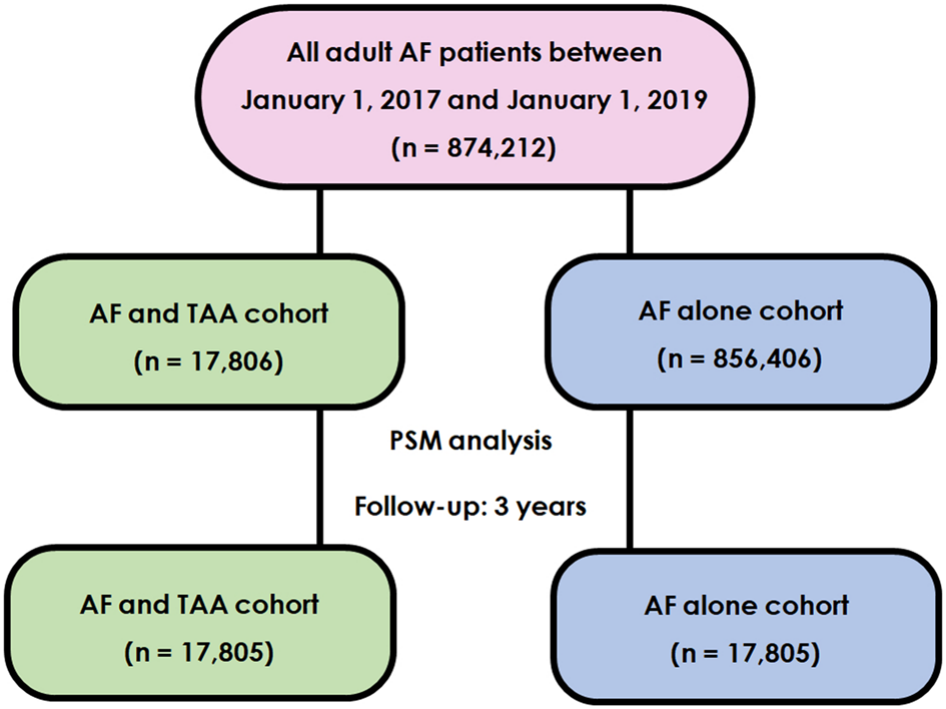
Study selection process

For the present study, the TriNetX research network was searched for the inclusion of AF patients (International Classification of Diseases, Tenth Revision, Clinical Modification [ICD-10-CM] code: I48) aged ≥ 18 years between 1 January 2017 and 1 January 2019. The cohort was placed into two groups based on the presence of AF and TAA with/without rupture (ICD-10-CM codes: I71.1 and I71.2, respectively) or AF alone (Fig. 1). In the TAA group, TAA should to have occurred in the 2017–2019 timeframe, whereas the group with AF alone should not have history ever of TAA. All diagnoses were identified by the ICD-10-CM codes in the EMRs. No other inclusion or exclusion criteria were defined. The searches were run in TriNetX on 22 February 2022. At the time of the search, there were 58 participating HCOs within the TriNetX research network.

Patients have been not involved in the design of the study; however, dissemination of the results is planned thorough patients’ associations.

### Follow-up and clinical outcomes

All patients were followed up from inclusion to at least 3-years. The primary endpoint was the occurrence of ischemic stroke/transient ischemic attack (TIA). All-cause mortality, major bleeding (composite of intracranial haemorrhage [ICH] and gastrointestinal bleeding) and the composite of any arterial or venous thrombotic event (any of the following: myocardial infarction, other arterial thrombosis, venous thromboembolism [VTE], or ischemic stroke/TIA, systemic embolism) were secondary outcomes.

### Statistical analysis

Continuous variables were expressed as mean and standard deviation (SD), and tested for differences with independent-sample *t* tests. Categorical variables were expressed as absolute frequencies and percentages, and tested for differences with chi-squared tests.

The TriNetX platform was used to run 1:1 propensity score matching (PSM) using logistic regression. The platform uses ‘greedy nearest-neighbour matching’ with a calliper of 0.1 pooled standard deviations and difference between propensity scores ≤ 0.1. Covariate balance between groups was assessed using standardised mean differences (SMDs). Any baseline characteristic with a SMD between cohorts < 0.1 is considered well matched [8].

Odds ratios (OR) with 95% confidence intervals (CI) were calculated following PSM. Hazard Ratios (HR) and 95% CI were also provided after PSM, as well as Kaplan–Meier survival curves with log-rank tests. No imputations were made for missing data. Two-sided *p* values < 0.05 were accepted as statistically significant. Statistical analysis was performed using the TriNetX Analytics function in the online research platform.

## Results

### Participant characteristics

Between January 2017 and 2019, 874,212 patients aged ≥ 18 years with AF were identified. Of these, 17,806 had a TAA with or without rupture accounting for an overall prevalence of 2.04%. Table 1 summarizes the baseline characteristics of patients with AF and TAA and patients without TAA, before and after PSM. Patients with AF and TAA had a higher risk profile with higher prevalence of comorbidities except for diabetes. After PSM, both cohorts were well balanced.

**Table 1:** Comparison of clinical characteristics of the study cohort before and after propensity score matching

	Initial populations					Propensity score matched populations				
	AF and thoracic aortic aneurysm *N* = 17,806		AF alone *N* = 856,406		SMD	AF and thoracic aortic aneurysm *N* = 17,805		AF alone *N* = 17,805		SMD
Age (years), mean (SD)	72.3	11.2	71.5	13.0	0.070	72.3	11.2	72.4	11.8	0.010
Male sex, *n* (%)	12,696	71.3	477,049	55.7	0.328	12,695	71.3	12,694	72.1	0.001
Comorbidities, *n* (%)										
Diabetes mellitus	4349	24.4	248,259	28.9	0.103	4349	24.4	4410	24.7	0.008
Hypertension	15,198	85.3	612,627	71.5	0.304	15,197	85.3	15,283	85.8	0.014
Heart failure	7431	41.7	257,004	30.0	0.246	7430	41.7	7385	41.4	0.001
Ischemic heart disease	10,096	56.7	330,475	38.5	0.368	10,095	56.6	10,139	56.9	0.001
Peripheral vascular disease	2764	15.5	71,955	8.0	0.220	2763	15.5	2676	15.1	0.014
Hyperlipidemia	12,221	68.6	462.091	53.9	0.304	12,220	68.2	12,411	69.7	0.023
Prior cerebrovascular disease	4940	27.7	160,439	18.7	0.214	4939	27.7	4972	27.9	0.004
Prior pulmonary embolism	1082	6.07	27,493	3.2	0.136	1081	6.1	1070	6.01	0.003
Chronic obstructive pulmonary disease	4077	22.8	135,998	15.8	0.178	4076	22.8	4069	22.8	0.001
Overweight/obesity	4526	25.4	173,090	20.2	0.124	4525	25.4	4520	25.3	0.001
Acute kidney failure and chronic kidney disease	6216	34.9	222,107	25.9	0.196	6215	34.9	6143	34.5	0.009
Diseases of liver*	2124	11.9	64,158	7.4	0.503	2124	11.9	2017	11.3	0.019
Neoplasms	6829	38.5	246,164	28.7	0.206	6828	38.3	6660	37.4	0.020
Pharmacological therapy, *n* (%)										
Beta-blockers	12,362	69.4	466,100	54.4	0.312	12,361	69.4	12,460	69.8	0.012
ACE inhibitors	6339	35.6	231,568	27.0	0.185	6339	35.6	231,568	36.0	0.009
Angiotensin II inhibitors	4112	23.1	141,734	16.5	0.164	4111	23.1	4102	23.0	0.001
Antilipemic agents	10,069	56.5	347,362	43.7	0.258	10,068	56.5	10,249	57.6	0.021
Calcium channel blockers	7871	44.2	281,116	32.8	0.235	7870	44.2	7815	42.1	0.006
Diuretics	9828	55.1	355,484	41.5	0.276	9827	55.1	9928	55.7	0.011
Antiarrhythmics	9586	53.7	331.972	38.7	0.303	9567	53.7	9616	54.0	0.006
Blood glucose regulation agents (including oral antidiabetics and insulin)	7546	42.3	283,048	33.1	0.193	7545	42.3	7511	42.2	0.004
Antiplatelets	10,347	58.1	356,904	41.6	0.332	10,346	58.1	10,417	58.5	0.008
Anticoagulants	12,575	70.6	477,618	55.8	0.311	12,574	70.6	12,671	71.2	0.012

*ACE* angiotensin-converting enzyme, *AF* atrial fibrillation, *SD* standard deviation, *SMD* standardised mean difference

^*^This includes K70-K77 International Classification of Diseases, Tenth Revision, Clinical Modification (ICD-10-CM) codes (K70 alcoholic liver disease, K71 toxic liver disease, K72 hepatic failure, not elsewhere classified, K73 Chronic hepatitis, not elsewhere classified, K74 fibrosis and cirrhosis of liver, K75 other inflammatory liver diseases, K76 other diseases of liver, and K77 liver disorders in diseases classified elsewhere)

### Ischemic stroke/TIA in patients with TAA and AF vs. those with AF alone

**Fig. 2:**
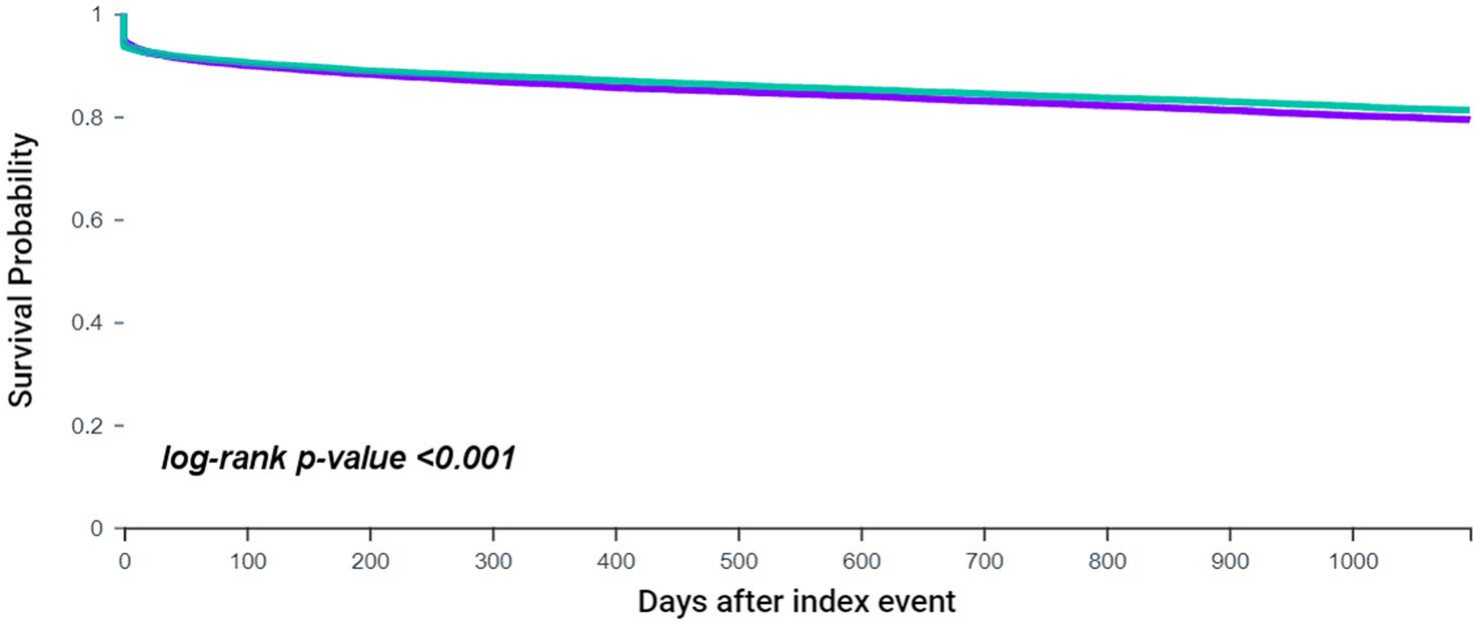
Kaplan–Meier curves showing survival free from ischemic stroke/TIA in patients with TAA and AF vs. those with AF alone

After PSM, 17,805 patients were included in each of the two cohorts (i.e. 1:1). During the 3-years of follow-up, 3079 (17.3%) AF patients with TAA and 2772 (15.6%) patients with AF alone developed an ischemic stroke/TIA. The risk of suffering from ischemic stroke/TIA was 1.13-fold higher in patients with AF and TAA (OR 1.13 95% CI 1.07–1.20), confirmed in the time-dependent analysis (HR 1.09, 95% CI 1.04–1.15; log-rank *p* value < 0.001) (Fig. 2).

### Secondary outcomes

After PSM, 6232 (35%) AF patients with TAA and 5872 (33%) patients with AF alone, suffered an arterial or venous thrombotic event during the 3-years of follow-up. For major bleeding, corresponding figures were 2500 (14%) and 2358 (13.2%), respectively; whereas for all-cause mortality they were 3027 (17%) and 3113 (17.5%). Of note, patients with AF and TAA had an increased risk of composite arterial or venous thrombotic events in comparison to patients with AF alone (OR 1.09, 95% CI 1.05–1.14; HR 1.05, 95% CI 1.01–1.09, log-rank *p *value = 0.004) (Supplementary Fig. 1). Regarding other secondary outcomes, the risk of major bleeding was higher in patients with AF and TAA (OR 1.07, 95% CI 1.01–1.14), but this was not significant in time-dependent analysis (HR 1.04, 95% CI 0.98–1.10; log-rank *p* value = 0.187). The risk of all-cause mortality was higher in patients with AF alone only in time-dependent analysis (OR 1.03, 95% CI 0.98–1.08; HR 1.06, 95% CI 1.01–1.12, log-rank *p* value = 0.017).

## Discussion

The principal findings of this analysis are as follows: (i) there is a clinical co-occurrence of TAA and AF; (ii) patients with AF and TAA are characterized by a higher cardiovascular risk profile, compared to those with AF and no history of TAA; and (iii) in PSM analysis, amongst patients with AF, TAA was associated with an increased risk of ischemic and systemic thromboembolic events at 3-year follow-up.

Emerging clinical evidence has shown a high prevalence of TAA among patients with AF. In a retrospective analysis from nationwide population database, Hsu et al. [3] reported a bidirectional association between aortic aneurysm and AF, showing that in patients with AF compared to those without AF, an increased incidence of aortic aneurysm was evident at 13 years follow-up (adjusted HR 1.24, 95% CI 1.10–1.40, *p* < 0.001). Similarly, patients with aortic aneurysm had a higher risk for presenting with AF at follow-up compared to patients without a diagnosis of aortic aneurysm (adjusted HR 1.187, 95% CI 1.079–1.301, *p* < 0.001) [3]. In a sub-analysis considering only TAA, a higher incidence was detected in patients with AF compared to those without (0.14% vs. 0.09%, *p* < 0.001) [3].

A cross-sectional study of patients with AF undergoing gated chest computer tomography performed as part of the assessment for pulmonary vein isolation, reported a TAA prevalence of 20%, with 1% of the TAA detected having a size approaching the current surgery indication [4]. From a pathophysiological perspective, atherosclerosis underlies TAA, and indeed, peripheral or coronary artery diseases are other common clinical manifestations of atherosclerosis. Both peripheral or coronary artery diseases are associated with incident AF and AF-related complications, and AF is a common complication after aortic procedures such as trans-catheter aortic valve replacement [9]. The findings regarding AF and TAA could be a non-casual association related to the increasing prevalence of both diseases with advancing age and consequently shared risk factors such as hypertension and heart failure. Indeed, the prevalence of TAA is approximately 4% in patients > 65 years and accounts for 6000 deaths a year in the UK [2]. Similarly, the prevalence of AF increases exponentially with age with an estimated ~ 6.9% prevalence in people > 65 years, though the burden of mortality linked to AF remains more elusive [10].

The finding in this study of a co-occurrence of TAA among patients with AF is aligned with previous results, though our figure being based on EMRs can include also AF post-surgery and therefore be an overestimate. While the associations may simply reflect shared risk factors, these findings raise the clinical implications regarding monitoring of patients with AF for the risk of developing TAA. Of note, the data show a higher prevalence of cardiovascular risk factors in patients with TAA and AF (but not diabetes) which may outline a more hemodynamic and degenerative nature of the TAA than an atherosclerotic origin.

Another major finding of this study is that patients with AF and TAA have an increased risk of stroke, TIA and systemic thromboembolic events compared to a matched AF population with a similar cardiovascular risk profile. Any attempt to provide a plausible mechanistic explanation for such a finding remains speculative although it may be hypothesized that the presence of an aneurysm may be linked to endothelial damage which is one pillar of Virchow’s triad [11]. Also, the presence of complex aortic plaque on the descending aorta is an independent risk factor for ischemic stroke in AF patients [5].

In a general population, the Aortic Plaques and Risk of Ischemic Stroke (APRIS) study [12] and the Stroke Prevention: Assessment of Risk in a Community (SPARC) [13] have recently questioned prior studies [14, 15], reporting a lack of association between the presence of a complex aortic plaque and risk of stroke at a general population level. On the other side, the association between TAA and aortic atherosclerotic plaque remains elusive while it has been shown for involvement of the abdominal and infra-renal aortic aneurysm [16].

In this analysis, there was an increased odd for major bleeding in the group with AF and TAA. The short-term follow-up we considered in this retrospective analysis may have biased this outcome, and the concomitant use of aspirin and/or OAC may have contributed to this. The increased mortality we detected in the group with AF alone compared to patients with AF and TAA which can be counterintuitive due to the well-known mortality linked to TAA, is possibly because patients with TAA of any size not requiring surgery were also included, as our search was based on ICD codes. Therefore, small thoracic aneurysms with slow rates of growth and no impact on survival have been potentially included.

### Clinical implications

We believe that the relevance of our finding is linked to the clinical perspective. Though this association seems a discordant comorbidity, AF and TAA have been shown to share commonalities in pathological pathways [17], the requirement of a CT scan to detect diseases of thoracic aorta has limited the applicability of screening program to general population and cannot be supported among AF for the risk benefit ratio considering the overall low figure of AF associated with TAA [17].

The added piece of the results of our analysis is that the coexistence of the two clinical conditions may confer a higher risk of adverse outcomes. Indeed, this claims attention for the need of optimizing the comprehensive medical management which may be difficult to integrate since the two diseases seem different. As a matter of fact, considering the higher cardiovascular risk profile of patients with AF and TAA, the proportion on antiplatelets appears to be high, while OAC is low, being 58.6 and 70.6%, respectively. This finding may be correlated to a perception from the surgeons of a bleeding risk which may lead to hold OAC notwithstanding the increased risk of stroke posed by AF. Similarly, considering the bulk of evidence showing that statins improve outcomes in both AF [18] and TAA [19], the proportion of patients on statins seems to be low. It may be hypothesized that the contradictory findings on the medical therapy prescribed may reflect an absence of an integrated management of both conditions.

### Limitations

Several limitations should be considered when interpreting the results of the current study. First, the participant information is based on EMRs, and from this, a distinction between pre-existing AF, new onset of AF and AF post-operative surgery cannot be made. This may explain the low proportion of patients on OAC therapy in the group with only AF, which is difficult to investigate further. Second, patients with thoraco-abdominal aneurysms were excluded in order to assess only the impact of TAA. Thirdly, information on the prevalence of complex aortic plaque in the two groups could not be recovered. In this study, the cohorts were PSM for several factors including age, sex, ethnicity and co-morbidities, but residual confounding factors may still be present and some health conditions may be underreported in EMRs. Finally, the analyses presented in this manuscript have been performed using the TriNetX platform which has the major limitations that data cannot be exported for the analysis. As a result, the graphical output of the software is not optimal and sometimes hinder a proper graphical appreciation of differences that are actually significant.

## Conclusion

Our retrospective analysis from a large global federated dataset reports a clinical concomitance of AF and TAA. Importantly, in a PSM analysis, an increased risk of ischemic events in patients affected by both TAA and AF was evident, compared to AF alone. Whether this association simply reflects shared risk factors or commonality in pathophysiological pathways, it raises relevant clinical implications that deserve further investigation.

## Supplementary Information

Below is the link to the electronic supplementary material.

Supplementary file1 (DOCX 174 KB)

## Data Availability

The data that support the findings of this study are available from TriNetX. To gain access to the data, a request can be made to TriNetX (https://live.trinetx.com), but costs may be incurred, and a data sharing agreement is needed.
