# Supplementary material for: Thoracic aortic aneurysm and atrial fibrillation: clinical associations with the risk of stroke from a global federated health network analysis
Source: Intern Emerg Med. 2023 Jan 14;18(2):423–8. doi: 10.1007/s11739-022-03184-6 (PMC10017617; doi:10.1007/s11739-022-03184-6)

**Supplementary Material**

**Supplementary Figure 1.** Kaplan-Meyer curves showing survival free from the secondary outcomes in patients with TAA and AF vs. those with AF alone.


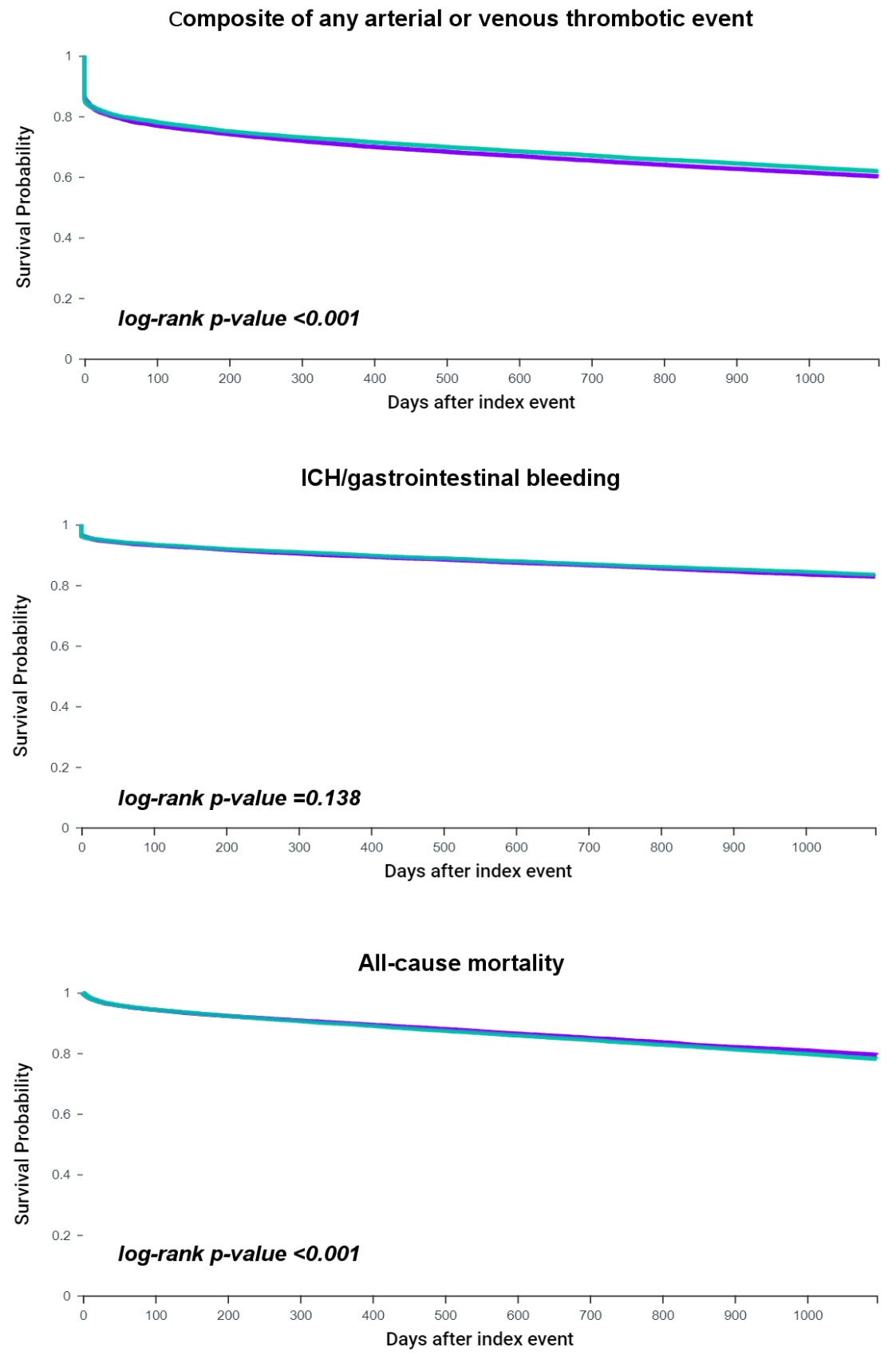

Supplement: Supplementary file 1 — Supplementary file1 (DOCX 174 KB) [file 11739_2022_3184_MOESM1_ESM.docx]
